# Increase in fatty acids and flotillins upon resveratrol treatment of human breast cancer cells

**DOI:** 10.1038/s41598-019-50416-5

**Published:** 2019-09-27

**Authors:** Luciana Gomes, Marcos Sorgine, Carlos Luan Alves Passos, Christian Ferreira, Ivone Rosa de Andrade, Jerson L. Silva, Georgia C. Atella, Claudia S. Mermelstein, Eliane Fialho

**Affiliations:** 1 Universidade Federal do Rio de Janeiro, Instituto de Nutrição Josué de Castro, Departamento de Nutrição Básica e Experimental, Rio de Janeiro, RJ 21941-590 Brazil; 2 Universidade Federal do Rio de Janeiro, Instituto de Bioquímica Médica Leopoldo de Meis, Rio de Janeiro, RJ 21941-590 Brazil; 3 Universidade Federal do Rio de Janeiro, Instituto de Ciências Biomédicas, Rio de Janeiro, RJ 21941-590 Brazil

**Keywords:** Breast cancer, Cellular imaging

## Abstract

Flotillin-1 and flotillin-2 are highly conserved proteins that localize into cholesterol-rich microdomains in cellular membranes. Flotillins are closely related to the occurrence and development of various types of human cancers. Flotillin-1 is highly expressed in breast cancer, and the high expression level of flotillin-1 is significantly correlated with poorer patient survival. Here we studied the relationship between the formation of lipid rafts and the expression of flotillins and lipids in human breast cancer cells. We used the polyphenol compound resveratrol to alter the structure and function of the plasma membrane. Our data revealed an increase in fatty acids in MCF-7 and MDA-MB-231 cells upon resveratrol treatment. Interestingly, we also found an increase in the expression of both flotillin-1 and flotillin-2 in breast tumor cells after treatment. Resveratrol also induced changes in the pattern of flotillin distribution among detergent-resistant lipid rafts fractions in both cell lines and induced the nuclear translocation of flotillin-2. Since resveratrol has been pointed out as a putative cancer therapy agent, our results could have an impact on the understanding of the effects of resveratrol in tumor cells.

## Introduction

Lipid rafts are specialized membrane structures that are involved in myriad of cellular functions, such as endocytosis, cell signaling and the control of cell survival and death. They are enriched in cholesterol, sphingolipids and specific proteins, such as flotillins. Several studies found that flotillins are closely related to the occurrence and development of tumors^1–3^. Flotillin-1 is highly expressed in breast cancer specimens, and the high expression level of flotillin-1 is significantly correlated with later clinical staging and poorer patient survival^1^. Knockdown of either flotillin-1 or -2 inhibits the proliferation and invasiveness of MCF-7 and MDA-MB-231 human breast cancer cells^1,4,5^.

Besides flotillins, alterations in lipids from membrane microdomains, are found in cancer cells, and they correlate with changes in the control of cell adhesion, cell proliferation, cell death, and cell signaling^6^. Thus, studies with substances that alter lipid rafts in cancer cells are relevant. It has been shown that resveratrol induces phase separation and formation of liquid-ordered domains in bilayer structures^7^. Resveratrol is a polyphenol compound present in grape skin and seeds, and it has been pointed out as a possible contributor to cancer therapy, antioxidant activity, cardiovascular protection, and neurodegenerative diseases treatment^8^. Noteworthy, the mechanism by which resveratrol exerts such pleiotropic effects remains unclear and controversial.

Resveratrol has a high affinity for the lipid part of membranes; the hydroxyl groups of resveratrol interact with the polar head groups of phospholipids^9^. Recently, it was found that resveratrol increases the surface area per lipid, decreases membrane thickness, and protects lipid membranes from hydrolysis by phospholipases^10^. Colin and colleagues showed a different interaction of resveratrol and the plasma membrane in cancer cells, where resveratrol could enter the cell by endocytosis via lipid rafts activating downstream signaling pathways^11^. They also showed that resveratrol promotes the redistribution of specific signaling proteins into lipid rafts, which could be associated with resveratrol anticancer effects. In face of the recent findings related to the influence of resveratrol in the organization of the plasma membrane, and specifically the lipid rafts, we decided to study the effects of resveratrol in the expression of flotillins and lipids in human breast cancer cells.

## Results

### Analysis of cell viability after resveratrol treatment

**Figure 1:**
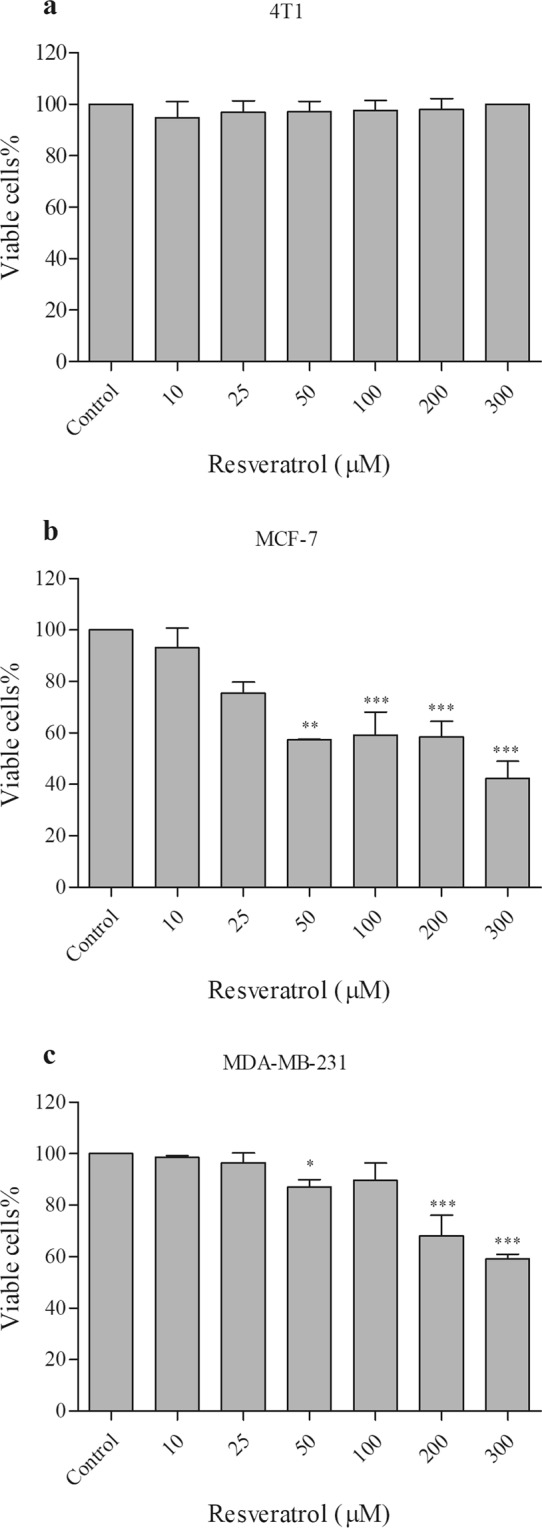
Effect of resveratrol on the cell viability of MCF-7 and MDA-MB-231 cells. (**a**) 4T1, (**b**) MCF-7 and (**c**) MDA-MB-231 cells (2 × 10^5^) were incubated in the presence of different concentrations (10 to 300 μM) of resveratrol, or 0.1% DMSO (control), for 24 h at 37 °C. They were then washed and maintained with MTT (0.5 mg/mL) for 3 h at 37 °C for formazan crystals formation and the associated optical density was analyzed at 570 and 650 nm. The results were expressed as mean ± SD, n = 3, *p < 0.05, **p < 0.01, ***p < 0.001 significantly different (p < 0.05) from the values corresponding to the control, using One-way ANOVA, followed by multiple comparison test Dunnett.

Here our aim was to study the effects of the lipid raft-inducer compound, resveratrol, in the expression of flotillins in human breast cancer cells. First, we investigated the effects of resveratrol treatment on the viability, using the MTT assay, of two human breast tumor cells (MCF-7 and MDA-MB-231) and in the non-tumor mouse breast epithelial cell line 4T1. We analyzed the viability of the cells after 24 hours of treatment using different concentrations of resveratrol (10, 25, 50, 100, 200 and 300 µM). No significant changes in cell viability was observed in non-tumor 4T1 cells (Fig. 1a).

As shown in Fig. 1, resveratrol induced cytotoxic effects on MCF-7 and MDA-MB-231 cells in a dose-dependent manner. Concentrations of resveratrol above 50 µM induced a significant decrease (~40–50%) in cell viability in MCF-7 cells, and a minor decrease (~10–40%) in cell viability in MDA-MB-231 cells (Fig. 1b,c). In MCF-7 cells, we observed a 43% reduction with 50 µM, 41% with 100 µM, 42% with 200 µM, and 58% with 300 µM (Fig. 1b). In MDA-MB-231 cells, we observed a 13% reduction with 50 µM, 10% with 100 µM, 32% with 200 µM, and 41% in 300 µM (Fig. 1c). Since the two cell lines presented different sensibilities to resveratrol in terms of cell viability, we decided to use 80 µM of resveratrol in all further experiments with MCF-7 cells and 200 µM of resveratrol in MDA-MB-231 cells.

### Isolation of lipid rafts

**Figure 2:**
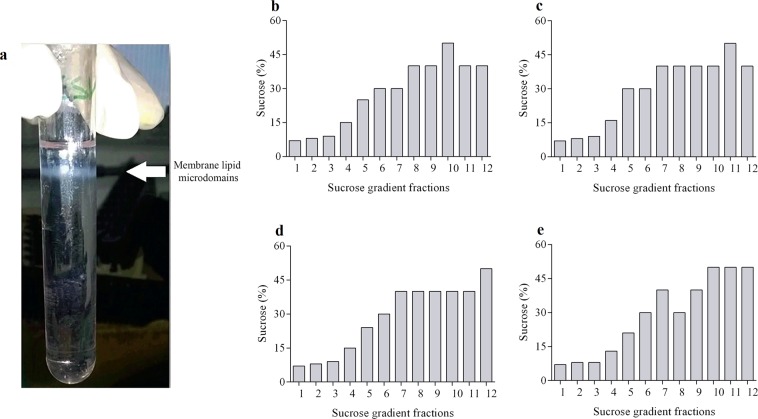
Isolation of membrane microdomains and analysis of the sucrose gradient profile after resveratrol treatment. MCF-7 and MDA-MB-231 cells (1 × 10^8^) after resveratrol treatment with 80 and 200 μM, respectively, or 0.1% DMSO (control) were sonicated (in TNE buffer) in the presence of different classes of proteases inhibitors. Afterwards, they were incubated with Triton X-100 detergent for 20 min at 4 °C. Subsequently, 80% sucrose solution was homogenized, followed by the addition of 30% sucrose and 5% sucrose solution, forming three distinct phases. The samples were centrifuged at 38,000 rpm for 22 h at 4 °C. Then divided into twelve 1 mL fractions. In each fraction, the refractive index was measured, which was converted to sucrose concentration. (**a**) A representative image of a tube with samples after centrifugation. The arrow indicates floating whitish material, visible in the median region of the tube, indicative of the presence of membrane lipid microdomains. Sucrose concentration profile on the gradient fractions of the (**b**) control, and (**c**) resveratrol-treated MCF-7 cells and, (**d**) control and (**e**) resveratrol-treated MDA-MB-231 cells.

To investigate the effects of resveratrol on lipid rafts, we decided to isolate lipid rafts from the two human breast tumor cell lines (untreated and resveratrol-treated cells) using a discontinuous sucrose density gradient centrifugation in the presence of Triton X-100 at 4 °C. Lipid rafts are insoluble in the nonionic detergent Triton X-100. At the end of the isolation procedure, it was possible to observe the formation of an intact lipid raft fraction floating on the top of the centrifuge tube in all the experiments (Fig. 2a). Twelve fractions were collected from each isolation procedure and the percentage of sucrose was determined in each one. We confirmed the formation of a sucrose gradient ranging from 5% to 50% in all four experimental conditions: untreated and resveratrol-treated MCF-7 and MDA-MB-231 cells (Fig. 2b–e).

### Resveratrol induces changes in the pattern of flotillin distribution among lipid rafts fractions

**Figure 3:**
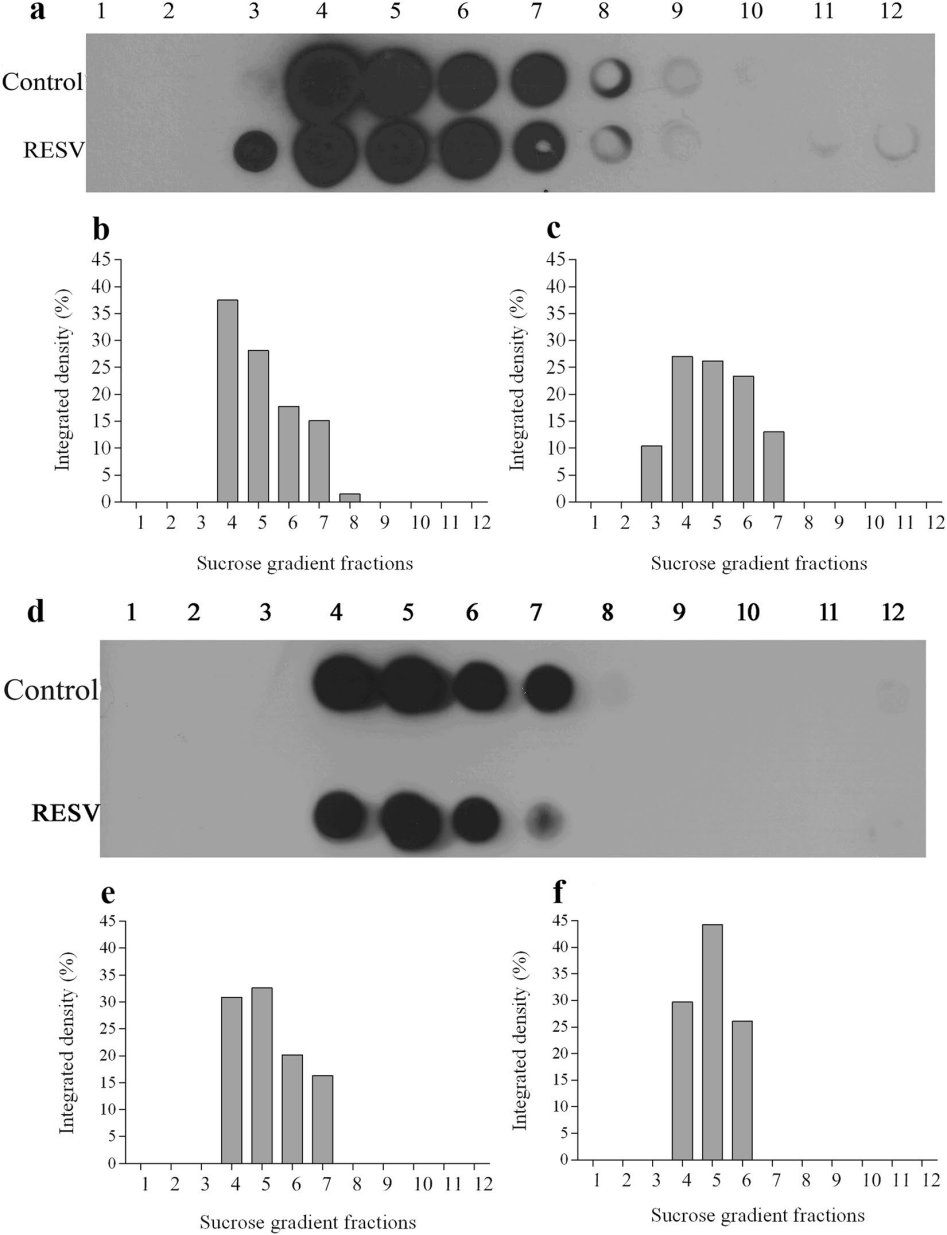
Resveratrol alters membrane lipid microdomain distribution in sucrose gradient fractions. A volume of 500 μL of each fraction was applied to the nitrocellulose membrane and were submitted to Dot blot analysis. Membrane was then blocked with TBS-Tween-Milk for 2 h at room temperature. Subsequently, it was incubated with anti-flotillin-1 antibody (1:800) in TBS-Tween-Milk for 18 h at 4 °C. Membrane was then washed and incubated with peroxidase-conjugated secondary antibody (1:1000) for 2 h at room temperature. The detection of flotillin-1 was performed by chemiluminescence. (**a**) MCF-7 and (**d**) MDA-MB-231cells. Densitometry, in percentage, of the scanned image for the samples (**b** and **e**) control (0.1% DMSO), and (**c** and **f**) resveratrol-treated cells.

Next, we decided to analyze the presence of flotillin proteins in the 12 fractions obtained in the lipid raft isolation since flotillins are considered lipid raft markers. Upon treatment of cells with resveratrol, lipid rafts were separated by sucrose gradient and then examined by dot blot analysis to assess the presence of flotillin-1 in both raft and non-raft fractions. Changes in the distribution of flotillin among the fractions were observed when comparing untreated with resveratrol-treated cells (Fig. 3). In resveratrol-treated cells, flotillins were found in fractions containing lower concentrations of sucrose as compared to control cells (Fig. 3). The results shown in Fig. 3 demonstrate that resveratrol treatment of MCF-7 and MDA-MB-231 cells resulted in a significant redistribution of the cellular flotillin-1 from the cytosol and plasma membrane (fractions 7–9) to the lipid raft microdomains (fractions 3–6).

### Resveratrol induces an increase in the expression of flotillins in breast cancer cells

**Figure 4:**
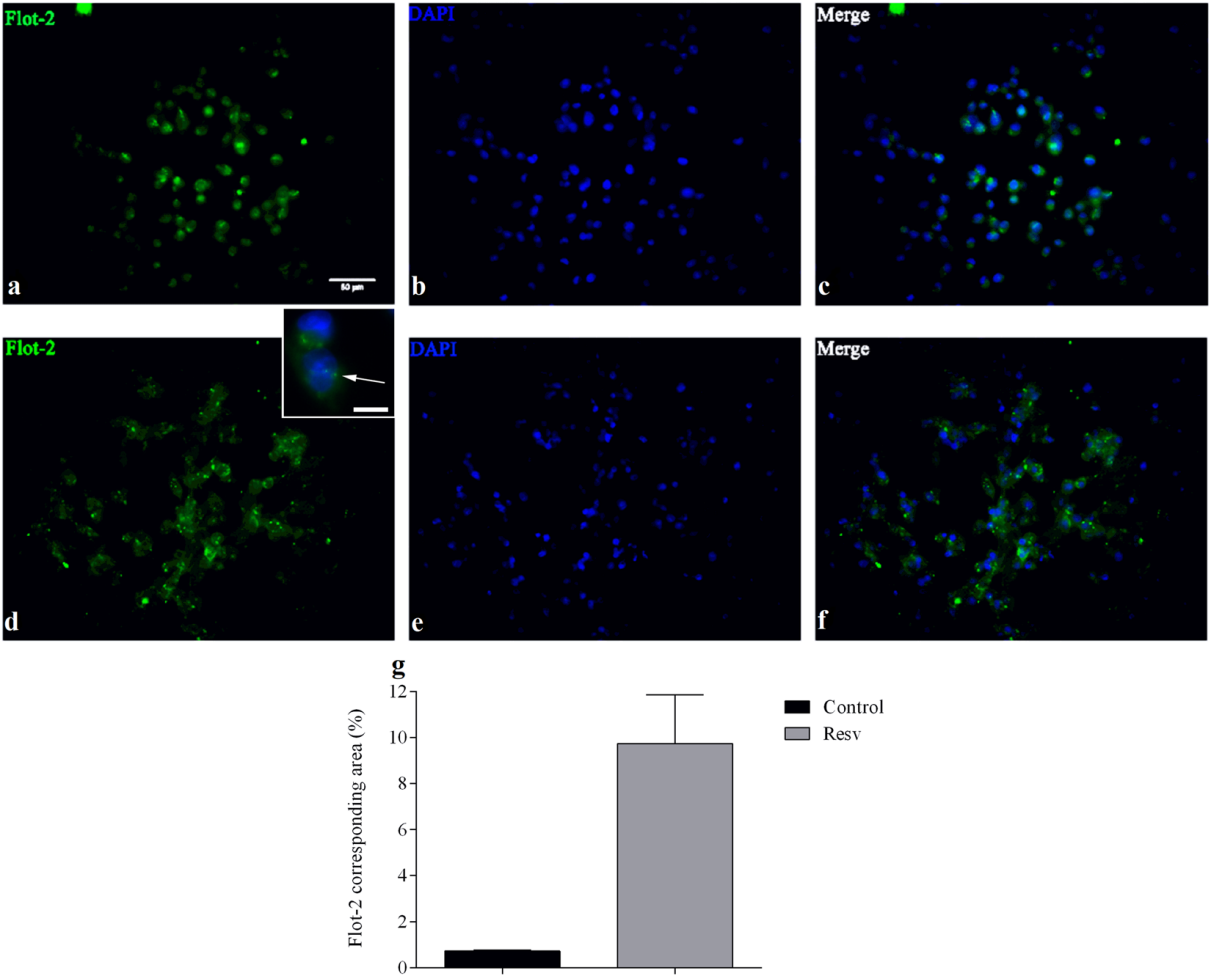
Immunofluorescence for flotillin-2 in MDA-MB-231 cells after resveratrol treatment. Cells (5 × 10^5^) were treated with 200 μM resveratrol or 0.1% DMSO (control) for 24 h. Then were washed with 0.5% PBS-Triton X-100, and incubated with anti-flotillin-2 antibody (1:70) for 1 h at 37 °C. Samples were then washed, and incubated with the secondary antibody (1:100) conjugated to the fluorophore for 1 h at 37 °C. Subsequently, cells were washed, and incubated with DAPI (1: 2,000) for 3 min. Images show the labeling for flotillin-2 (green) in control cells (**a**) and treated cells (**d**). The nuclei in the control (**b**) and treated (**e**) samples were stained with DAPI (blue). (**c**,**f**) are the overlap of the images of the control samples (**a**,**b**) and treated (**d**.**e**), respectively. (**g**) representation in percentage of the area corresponding to flotillin-2. The results were expressed as mean ± SD, n = 5 for the control sample and n = 9 for the treated sample. Bar in (**a**) = 50 µm and bar in the inset = 10 µm. The arrow in the inset points to flotillin-2 dots.

Since we detected changes in the distribution of flotillins among rafts and non-rafts fractions after treatment with resveratrol (Figs 2 and 3), we decided to analyze the expression of flotillin proteins in untreated and resveratrol-treated cells using immunofluorescence microscopy. MDA-MB-231 cells were treated with resveratrol, fixed and labeled with anti-flotillin-2 antibodies. Immunofluorescence microcopy analysis revealed a 10-fold increase in the expression of flotillin-2 in treated cells as compared to control (Fig. 4). It is important to point out that treatment of MDA-MB-231 cells with 200 uM resveratrol induced a 32% decrease in cell viability (Fig. 1c), and nevertheless we found a 10-fold increase in the labeling of flotillin-2 in these cells.

**Figure 5:**
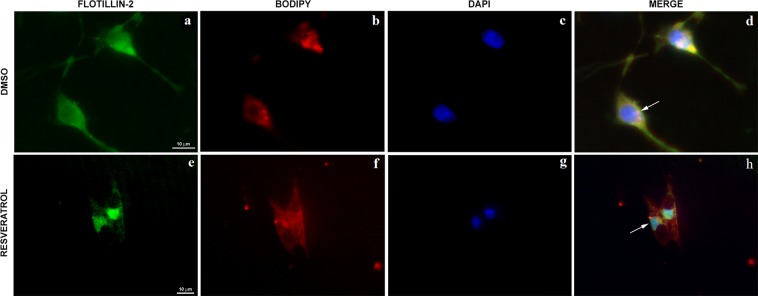
Resveratrol effects on the distribution of flotillin-2 in MDA-MB-231 cells. Cells were treated with 200 μM resveratrol for 24 h, triple-labelled with an antibody against flotillin-2 (**a**,**e**), Bodipy-ceramide (a Golgi specific fluorescent probe, B and F) and DAPI (**c**,**g**) and analyzed under a Spinning Disk confocal microscope. Merged images are shown in (**d**,**h**). Arrow in (**d**) points to flotillin-2 in Golgi membranes and arrow in (**h**) points to flotillin-2 within the nuclei of cells. Bars in (**a**,**e**) = 10 µm.

To further study the subcellular localization of flotillin-2 in control and resveratrol-treated cells, we triple-labelled MDA-MB-231 cells with Bodipy-ceramide (a Golgi specific fluorescent probe), flotillin-2 and DAPI and analyzed the cells under a Spinning Disk confocal fluorescence microscope (Fig. 5). Interestingly, we found that flotillin-2 colocalizes with Golgi membranes in control MDA-MB-231 cells (DMSO) but translocate to the nuclei after resveratrol treatment (Fig. 5).

**Figure 6:**
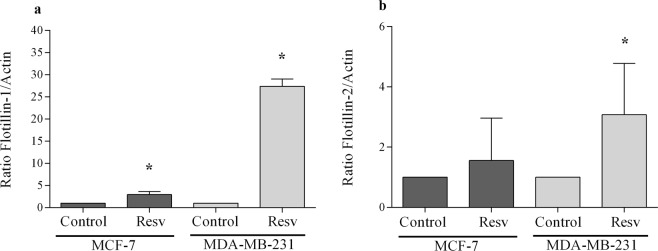
Resveratrol effect on the mRNA expression of flotillin-1 and -2 in MCF-7 and MDA-MB-231 cells. MCF-7 and MDA-MB-231 cells were treated with 80 and 200 μM resveratrol, respectively, or 0.1% DMSO (control), for 24 h. Subsequently, the RNA was extracted using the Trizol reagent. Two micrograms of total RNA were used in cDNA synthesis. The qPCR was performed with flotillin-1 (**a**), and flotillin-2 (**b**) primers, and the results obtained after normalization as a function of actin expression, showed as mean ± S.D., n = 3, *p < 0.05.

**Figure 7:**
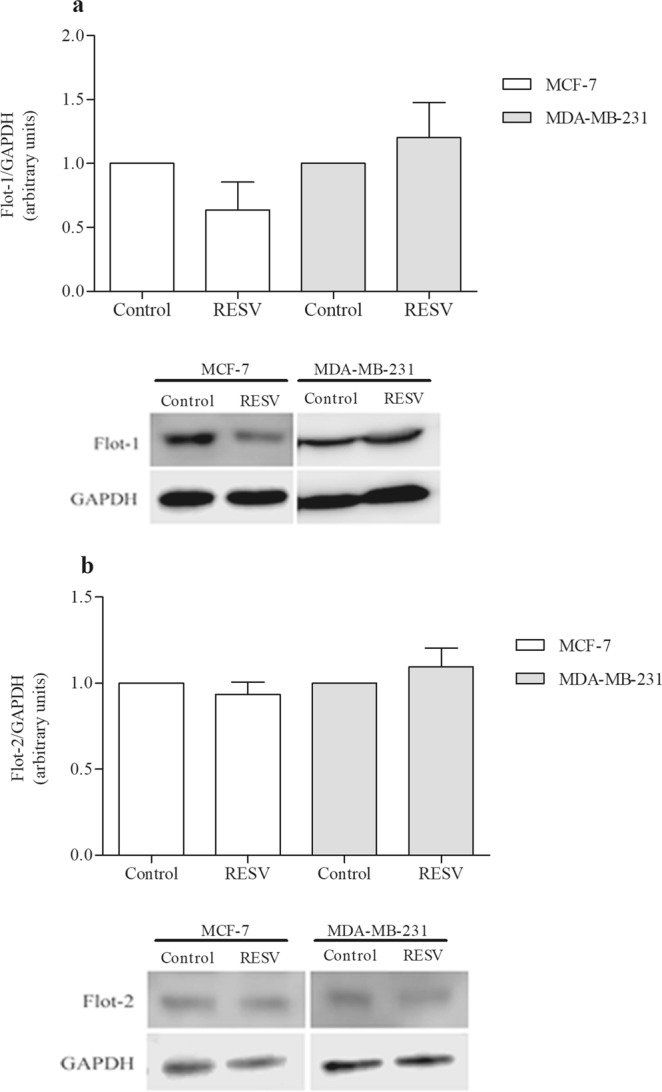
Resveratrol effect on flotillin-1 (**a**) and -2 (**b**) in MCF-7 and MDA-MB-231 cells at the protein level. MCF-7 and MDA-MB-231 cells after resveratrol treatment with 80 and 200 μM, respectively, or 0.1% DMSO (control), were submitted to Western blot analysis (80 μg of total protein extract) under denaturing conditions. The values were expressed in arbitrary units, showed as mean ± S.E, n = 3, n.s.: non-significant.

We also investigated the expression of flotillins at both the mRNA and protein levels in MCF-7 and MDA-MB-231 cells after treatment with resveratrol. The results of qPCR showed that resveratrol treatment induces an increase in the expression of flotillin-1 and -2 (Fig. 6), with the only exception of the expression flotillin-2 protein in MCF-7 cells, which showed no significant alterations. Western blots showed no significant changes in the expression of both flotillin-1 and -2, at the protein level, in MCF-7 and MDA-MB-231 cells after resveratrol treatment (Fig. 7). Interestingly, a slight increase in the levels of both flotillin-1 and -2 can be observed in MDA-MB-231 cells after resveratrol treatment, but not in MCF-7 cells (Fig. 7).

### Isolation of neutral lipids

**Figure 8:**
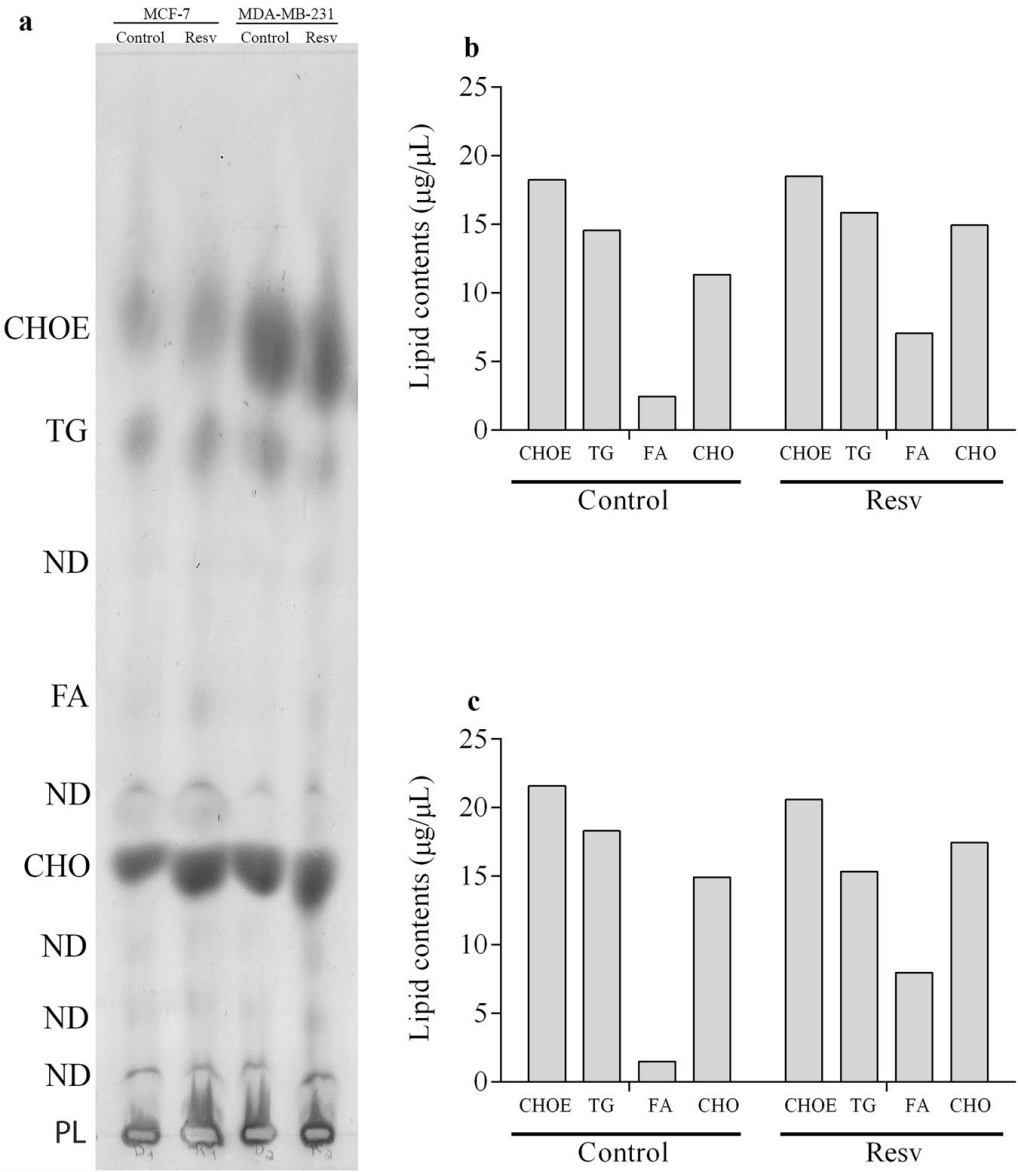
Resveratrol alters the neutral lipid profile in MCF-7 and MDA-MB-231 cells. Cells (1 × 10^7^) after resveratrol treatment, or 0.1% DMSO (control), for 24 h, were submitted to lipid extraction. Subsequently, the entire contents of total lipids extracted were subjected to TLC, in a solvent set consisting of n-hexane:diethylether:acetic acid (60:40:1 v/v). After evaporation of the solvents, (**a**) TLC was revealed with the Charring reagent and heating at 200 °C. CHOE (cholesterol ester), TG (triacylglycerol), FA (fatty acids), CHO (free cholesterol) content for (**b**) MCF-7 cells, and (**c**) MDA-MB-231 cells were expressed as μg/μL, after normalization with the respective standards.

Then we decided to investigate whether the changes in flotillins expression and in lipid rafts observed after resveratrol treatment were accompanied by changes in neutral lipids. Neutral lipids were isolated from the same initial number of MCF-7 and MDA-MB-231 cells (10^7^ cells) and we quantified the amounts of fatty acids (FA), triacylglycerol (TG), cholesterol ester (CHOE), and cholesterol (CHO) in MCF-7 and MDA-MB-231 untreated and treated with resveratrol (Fig. 8). Interestingly, we found a significant increase in the total amount of fatty acids (FA) in both cell lines (MCF-7 and MDA-MB-231) after resveratrol treatment: an 188% increase in MCF-7 cells and an 435% increase in MDA-MB-231 cells (Fig. 8).

## Discussion

Our study analyzed the effects of resveratrol treatment in the expression and distribution of flotillins and neutral lipids in human breast cancer cells. We found that resveratrol induced: (I) a cytotoxic effect in a dose-dependent manner; (ii) an increase in the expression of flotillin-1 and -2; (iii) an increase in the total amount of fatty acids; and (iv) a redistribution of flotillin-1 from the cytosol and plasma membrane to lipid raft microdomains and the nuclear translocation of flotillin-2.

In relation to the cytotoxic effects, we found that concentrations of resveratrol above 50 μM induced a significant decrease in cell viability in MCF-7 and in MDA-MB-231 cells, although MDA-MB-231 cells were found to be more resistance to treatment. These results show that MCF-7 cells are more sensitive to resveratrol than MDA-MB-231 cells, which are in accordance with a previous work^12^. MDA-MB-231 is a triple-negative type of human breast cancer (TNBC) cell line and consider to be a highly aggressive type of cancer cell, whereas MCF-7 expresses estrogen receptor and is a less aggressive cancer cell.

The increased sensitivity in cell viability of MCF-7 cells to resveratrol suggests that resveratrol action could be mediated, at least in part, by the estrogen receptor found in these cells. More studies are necessary to investigate the molecular details of the interaction between resveratrol and membrane receptors in MCF-7 cells. Importantly, no significant changes in cell viability was observed in the non-tumor breast cell line 4T1 reinforcing the idea that breast tumor cells are more sensitive to resveratrol than non-tumor breast cells.

Interestingly, we found an increase in fatty acids and in flotillin-1 and -2 in MCF-7 and MDA-MB-231 cells upon resveratrol treatment. Flotillins and fatty acids are both essential components for the formation of lipid rafts and therefore our results are in accordance with a previous work showing that resveratrol induces phase separation and formation of liquid-ordered domains in bilayer structures^7^. Favoring this hypothesis, we found a significant increase in the total amount of fatty acids, but not in cholesteryl ester and triacylglycerol, in both cell lines (MCF-7 and MDA-MB-231) after resveratrol treatment. It has been shown that lipid rafts are rich in phospholipids and cholesterol, whereas contain small amounts of cholesteryl ester and triacylglycerol^13^.

Our data also showed that resveratrol induced changes in the pattern of flotillin distribution among detergent-resistant lipid rafts fractions in both cell lines. Resveratrol treatment of MCF-7 and MDA-MB-231 cells resulted in a significant redistribution of the cellular flotillin-1 from the cytosol and plasma membrane to lipid raft microdomains.

Curiously, we found a 10-fold increase in the immunofluorescence labeling of flotillin-2 in MDA-MB-231 cells treated with 200 uM resveratrol; the same concentration of resveratrol which induced a 32% decrease in cell viability in this cell line. These data suggest a correlation between the concentration of resveratrol treatment and the expression of flotillin and cell viability: high resveratrol concentrations induce high flotillin expression and low cell viability in MDA-MB-231 cells. These results could be correlated with the nuclear translocation of flotillin-2 that we found after resveratrol treatment. Nuclear flotillin-2 could have a role in the regulation of the expression of genes related to cell survival and cell proliferation, which are hallmarks of cancer cells. In accordance with our results and hypothesis, the nuclear translocation of flotillins has been previously correlated with mitogenic activity in the human prostate cancer cell line PC-3^14^.

Curiously, only few papers describe the presence of flotillin-2 within the nuclei of cancer cells, and therefore nuclear flotillin-2 cannot be described as a characteristic of all cancer cell types or specific to breast tumor cancer cells. Further studies are necessary to unravel the role of nuclear flotillin-2 in different cancer cell types.

Finally, we found flotillin-2 within vesicle-like compartments after resveratrol treatment. These results suggest that upon resveratrol treatment flotillins are upregulated and they accumulate in intracellular compartments before being directed to the nucleus or to lipid rafts at the plasma membrane. It has been shown by different groups that flotillins display a dynamic cellular localization and they can be found in four major locations in the cells: plasma membrane, nucleus, endosomal structures and/or intracellular vesicles^15^. The molecular regulation of the dynamic changes in the cellular distribution of flotillins after resveratrol treatment needs to be further studied.

Triple-negative MDA-MB-231 cells exhibit a high level of resistance to chemotherapeutic drugs^16,17^. Chemoresistance has a range of causes, which includes the following: highest expression of phosphorylated protein kinase D isoform 2 (PKD2), activity of ATP-binding cassette (ABC) transporters, such as P-glycoprotein1 (MDR1 and ABCB1), ABCG2 and CCL20, which function as drug efflux pumps^16,18^.

One main question that arises from our work is how resveratrol induces flotillin expression. The expression of both flotillins involves transcriptional regulation and can be modulated by different transcription factors^19^. Flotillins are transcriptional targets of the extracellular signal-regulated kinases (ERK1/2) and the transcription factor Egr1 and serum response factor (SRF), both of which are ERK1/2 downstream targets, were identified as positive regulators of flotillin expression. Interestingly, it has been shown that ERK1/2 accumulates in lipid rafts on resveratrol exposure and could enter the cell by endocytosis via lipid rafts activating ERK1/2 downstream signaling pathways^11^. Here, we hypothesize that the expression of flotillin genes could be activated by ERK1/2 signaling pathway after resveratrol treatment.

Since resveratrol has been pointed out as a putative cancer therapy agent, our results could have an impact in the understanding of the effects of resveratrol in different human breast tumor cells.

## Materials and Methods

### Cell cultures

The human mammary gland/breast epithelial cell lines MCF-7 and MDA-MB-231, and the mouse mammary gland/breast epithelial cell line 4T1 were obtained from American Type Culture Collection (ATCC® HTB-22™, HTB-26™ and CLR-2539™ respectively). Cells were grown in DMEM (Dulbecco’s Modified Eagle’s medium) containing 10% fetal bovine serum and 1% penicillin and streptomycin solution (Sigma-Aldrich, USA), in a humidified 5% CO_2_ atmosphere at 37 °C. For treatment, cells were cultured up to 70–80% confluences and then were treated with  10–300 µM of trans-resveratrol (Sigma-Aldrich, USA), for 24 h. 0.1% DMSO (Dimethyl sulfoxide, Sigma-Aldrich, USA) was used as control.

### Cell viability assay

Cell viability was determined using 3-(4,5-dimethyl-2-thiazyl)-2,5-diphenyl-2H-tetrazolium bromide (MTT) reagent (Sigma-Aldrich, USA). Briefly, cells were plated at an initial density of 2.0 × 10^5^ cells per well in 24-well plates and incubated for 24 h at 37 °C under 5% CO_2_. Cultures were treated with resveratrol (10 to 300 μM) for 24 h. Cells treated with 0.1% DMSO were used as control. After 24 h of treatment, the supernatant of each well was removed, and cells were washed twice with PBS. Then, 500 µL MTT solution (5.0 mg/mL in PBS) were added and cells were incubated for 3 h at 37 °C, 5% CO_2_. The resultant formazan crystals were dissolved in isopropanol (500 µL) and absorbance intensities were measured in a microplate reader (FlexStation® 3 Reader, USA) at 570 nm and 650 nm^20^. All experiments were performed in triplicates, and the relative cell viability (%) was expressed as a percentage relative to the control cells.

### Detergent-resistant membrane purification and Dot blot

 Cells were washed in phosphate buffered saline (PBS, pH 7.2), and TNE buffer (100 mM Tris, 150 mM NaCl, and 0.2 mM EDTA; pH 7.5) was added together with 2 µL protease inhibitor cocktail 10 times diluted (Sigma-Aldrich, USA). Aliquots of 1 × 10^8^ cells in TNE buffer were carefully disrupted on ice with a Branson Sonifier Cell disruptor 250 (Analog Ultrasonic Homogenizer, USA) operating at 10% of total amplitude, with 3 cycles of 10 s and with 1 s rest between cycles, yielding a total homogenate. This homogenate was incubated for 20 min at 4 °C with 2% Triton X-100 in TNE buffer. After incubation, the sample was mixed 1:1 with 80% sucrose (w/v) in TNE buffer and transferred to a Beckman SW41 centrifuge tube (Beckman Coulter Inc, USA). This mixture was first overlaid with 30% sucrose, followed by 5% sucrose, and centrifuged at 38000 rpm for 20 h at 4 °C. Twelve 1-ml fractions could be sequentially collected from the top. Anti-flotillin-1 dot blotting - 0.5 mL from each gradient fraction was adsorbed onto a nitrocellulose filter using a dot-blot apparatus (GE Healthcare Life Sciences Corp., UK). Membranes were blocked for 2 h in Tris-buffered saline containing 1% Tween 20 (TBS-T) and 5% nonfat milk and incubated for 2 h with the primary antibody (1:1000), then washed with TBS-T and incubated with a peroxidase-conjugated secondary antibody (1:1000) for 2 h. Antigen-antibody complex formed were visualized with the enhanced chemiluminescence (ECL) kit (Amersham, UK) using Kodak X-Omat films (Eastman Kodak Comp., USA). Images were analyzed using IMAGE J 1.50i software (NIH, USA) and results were expressed as arbitrary units.

### Western blot

For preparation of protein extracts, after treatment for 24 h, cells were washed with PBS and lysed in liquid nitrogen. Cells were then scraped using lysis buffer (5 mM Tris-HCl, 10 mM ethylenediamine tetraacetic acid, 5 mM sodium fluoride, 1 mM sodium orthovanadate, 1 mM phenylarsine oxide, 1 µM okadaic acid, and 1 mM phenylmethylsulfonyl fluoride; pH 7.4), and protease inhibitor cocktail (Sigma-Aldrich, USA). The lysate was collected, sonicated, and cleared by centrifugation for 5 min at 4 °C and supernatant was stored at −80 °C. Equal amounts of total cellular proteins (80 µg) were used on sodium dodecyl sulfate-polyacrylamide gel electrophoresis (SDS-PAGE) and transferred onto polyvinylidene difluoride (PVDF) membranes (Immobilon P, Millipore, USA). Membranes were blocked for 2 h in Tris-buffered saline containing 1% Tween 20 (TBS-T) and 5% nonfat milk and incubated for 2 h with the primary antibody (1:1000), then washed with TBS-T and incubated with a peroxidase-conjugated secondary antibody (1:1000) for 2 h. Protein bands were visualized with the enhanced chemiluminescence (ECL) kit (Amersham, UK) using C-DiGit Chemiluminescent Western Blot Scanner (LI-COR Biotechnology, USA). Images were analyzed using Image J 1.50i software (NIH, USA), and results were expressed as arbitrary units, calculated by the fraction of pixels presented by each band relative to the GAPDH bands.

### Immunofluorescence microscopy

 MDA-MB-231 cells were cultivated on coverslips with 0.1% poly-L-lysine, and treated with 200 µM resveratrol, and 0.1% DMSO (control) for 24 h. Then, cells were rinsed with PBS and fixed with 4% methanol for 15 min at room temperature. They were then permeabilized with 0.5% Triton-X 100 in PBS 3 times for 10 min. The same solution was used for all subsequent washing steps. Cells were incubated with primary antibodies for 1 h at 37 °C. After incubation, cells were washed for 30 min and incubated with Alexa Fluor-conjugated secondary antibodies for 1 h at 37 °C, and nuclei were labeled with DAPI (0.1 μg/mL in 0.9% NaCl). Some cells were triple-labelled with Bodipy-ceramide (Thermo Fisher Scientific, USA), flotillin-2 and DAPI. Bodipy-ceramide was used for labeling Golgi with a fluorescent probe. Cells were examined either with an Axiovert 100 microscope (Carl Zeiss, Germany) or with a Spinning Disk confocal microscope (Olympus, Japan). Image processing was performed using Fiji software (based on ImageJ, http://imageJ.nih.gov/ij/).

### qPCR

Real-time quantitative polymerase chain reaction (qPCR) was performed in untreated and resveratrol-treated cells. mRNA from cells were isolated using Trizol (Invitrogen, USA) reagent, according to the manufacturer’s instructions, and quantified using a Nanodrop spectrophotometer. Two micrograms of total RNA were used as a template for cDNA synthesis, using the High Capacity cDNA Reverse Transcription kit (Life Technologies, USA). PowerUpTM SYBR® Green Master Mix (Applied Biosystems, USA), was used to quantify mRNA expression levels, with actin as an endogenous control. Real-time reactions were performed in triplicates using a StepOne Real-Time PCR Systems (Applied Biosystems, USA). Relative quantification was performed using the Delta-Delta Ct method. Primer sequences were as follows:

Actin_F: GTCCGCGATATCAAGGAAAA

Actin_R: GTGTTGGCGTACAGGTCCTT

Flotillin-1_F: CTCCACCCCACCTCAACTTATTTA

Flotillin-1_R: TCCAGCCCATCCCTCAGTCT

Flotillin-2_F: CCCCAGATTGCTGCCAAA

Flotillin-2_R: TCCA-CTGAGGACCACAATCTCA

### Lipid extraction

After treatment with resveratrol, 1.0 × 10^7^ cells were washed twice with PBS (Phosphate-buffered saline) (pH 7.5). The lipids were extracted as described previously by Bligh and Dyer^21^, methanol:chloroform:water (2:1:0.8 v/v) solution was added to the cells, they were, then, vortexed for 1 hour and centrifuged at 4 °C for 20 min at 3000 rpm. The lipid-containing supernatants were separated from the pellet. This process was performed twice, and the supernatants were united. The supernatants were added to water:chloroform (1:1) and vortexed for 30 s. The samples were centrifuged (3000 rpm/30 min at 4 °C), then the lipid phase was separated, and the solvent was evaporated by an N_2_ stream. Lipids extracts were resuspended in chloroform for analysis by TLC and HPTLC.

### Neutral lipids analysis

Lipid extracts used for neutral lipids were analyzed by one-dimensional thin layer chromatography (TLC) and high performance thin layer chromatography (HPTLC) on Silica plate (Merck, Germany). After applying the samples on the plate, the run was performed with n-hexane:ethylether:acetic acid (60:40:1 for TLC and 30:20:05 for HPTLC; v/v)^22^. Cholesterol (CHO), cholesterol ester (CHOE), triacylglycerol (TG) and fatty acid (FA) (Sigma-Aldrich, USA) standards were running in parallel to the samples to enable identifications and quantification of the bands. The lipids were visualized using a charring reagent after heating at 200 °C for 5 min^23^. After that, the chromatography plates were digitalized and analyzed using Image J 1.50i software (NIH, USA). The results were expressed as µg/µL, calculated by the fraction of pixels presented by each band relative to the respectively standard lipids.

### Statistical analysis

All the values were represented as the means ± standard error. Statistical analysis was performed with unpaired t-test and one-way ANOVA, followed by multiple comparison tests Tukey, Sidak and Dunnett and statistical significance was defined as *p < 0.05, **p < 0.01, ***p < 0.001.
